# State Estimation of Axisymmetric Target Based on Beacon Linear Features and View Relation

**DOI:** 10.3390/s21175750

**Published:** 2021-08-26

**Authors:** Xiaohua Cao, Shuaiyu Peng, Daofan Liu

**Affiliations:** School of Logistics Engineering, Wuhan University of Technology, Wuhan 430063, China; pengshy@whut.edu.cn (S.P.); ruheldf@whut.edu.cn (D.L.)

**Keywords:** axisymmetric targets, state estimation, linear features, view relation

## Abstract

In order to realize state estimation for axisymmetric targets and improve the accuracy and robustness of state estimation, a state estimation method for axisymmetric targets based on beacon linear features and view relation is proposed in this paper. The depth camera is used to collect the image and depth information of the object, and the features of the beacon line are extracted by the thinning process and Hough transform. Then, the rotation matrix model based on view relation is constructed to solve the target state. Finally, an axisymmetric shore power plug is taken as the experimental object and the L–V (linear features and view relation) state estimation method is compared with the C–H and C–IPPE state estimation methods. The experimental results show that the L–V state estimation method has higher accuracy and robustness.

## 1. Introduction

In the visual guidance system of the manipulator in industrial production, an effective target position and attitude are important for the manipulator to realize grasping, sorting, assembling, and other operations. The target pose estimation based on monocular vision is a process of calculating the spatial position and attitude of the 3D target object according to the two-dimensional image. At present, the features used in target state estimation are mainly 2D image features [1] and 3D reconstructed point cloud features [2,3]. According to the different models and algorithms, it can be divided into traditional feature–based state estimation and deep–learning–based state estimation [4,5]. Among them, traditional feature–based state estimation can be further subdivided into the target state estimations based on feature points, feature lines, template matching, and 3D features.

The state estimation based on feature points is to solve the Perspective–N–point (PnP) problem [6]. In this problem, the co-ordinates of [*n*] (*n* ≥ 3) feature points in the world co-ordinate system, the pixel co-ordinates in the image, and camera internal parameters are known. The co-ordinates of the target in the camera co-ordinate system are solved by using the perspective relation and the above conditions. Such a method is divided into two parts, feature points extraction and state solving method:

(1) Feature points extraction. For objects with obvious texture features, classic SIFT [7], SURF [8], ORB [9] methods, and so on can be used to complete the extraction of feature points. For the objects with unobvious texture features, it is often necessary to mark the feature points manually to achieve the purpose.
(2) State solving method. The solving method can be divided into an iterative method and noniterative method [10]. The iterative method is to construct the objective function of minimizing the residuals of the image square, and then to obtain the optimal state solution through a Newton method [11] or Levenberg–Marquardt (LM) [12] algorithm. However, this algorithm involves many parameters and has low computational efficiency. Among the noniterative algorithms, P4P, P5P, and other algorithms establish the linear equations between the three-dimensional points and the pixel points, that is, the method for solving the homography matrix (C–H method) [13,14]. In addition, Toby Collins et al. [15] proposed a C–IPPE state estimation method, whose solution method is very fast and allows people to fully characterize the method in terms of degeneracies, number of returned solutions, and the geometric relationship of these solutions. In addition, the C–IPPE method is more accurate than the PnP methods in most cases. However, the C–H and C–IPPE state estimation methods based on feature points are extremely dependent on the accuracy of point detection and the accuracy of point pairs matching, and the error of corner detection will directly lead to the deviation of state calculation and the deterioration of robustness.

The state estimation based on line features is mainly for the weak texture objects with few feature points, where it is difficult to establish the relationship between feature points and state using feature points. Moreover, the robustness of the line is better than that of the point, which is less affected by light and noise. Bin Wang et al. [16] proposed an attitude measurement algorithm suitable for aircraft targets, which made full use of the advantages of high accuracy, good stability, and strong antiocclusion ability of linear feature extraction, and the attitude error angle could reach within 1°. Yunxi Xu et al. [17] proposed an extended orthogonal iterative algorithm to realize attitude estimation based on the features of points and line segments. However, in both of these, multiple linear features are used, which makes the application scope of the algorithm have certain limitations. The noniterative linear solution method based on the linear feature is often not accurate enough, while the iterative method has high accuracy but poor real–time performance.

The state estimation based on template matching is mainly based on the 3D model of the object to establish the image template library of different states. By matching the real object’s state with the image in the model library, if the matching error is less than a certain threshold, the 3D model’s state is taken as the estimated value of the real object’s state. Bing Ji et al. [18] estimated the attitude angle of an aircraft by using the dynamic model matching principle, and proposed the cross–search method to accelerate the optimal model matching speed according to the distribution law of the similarity matrix. This method requires a high–precision model, a large number of data sets, and an efficient search and matching algorithm.

The state estimation based on 3D features, with the popularity of an RGBD sensor, is transformed from 2D–3D data point pairs to 3D–3D data point pairs. Without the auxiliary equipment, which is necessary for the monocular camera to estimate the state of the target, the depth feature of the pixel can be measured quickly and conveniently. Among them, the pose estimation based on 3D point cloud features [19,20] can ignore textures, but its algorithm is complex. In addition, for highly axisymmetric objects, it is difficult to detect the state angle around the axis of symmetry, as in the example of the shore power plug mentioned below.

**Figure 1:**
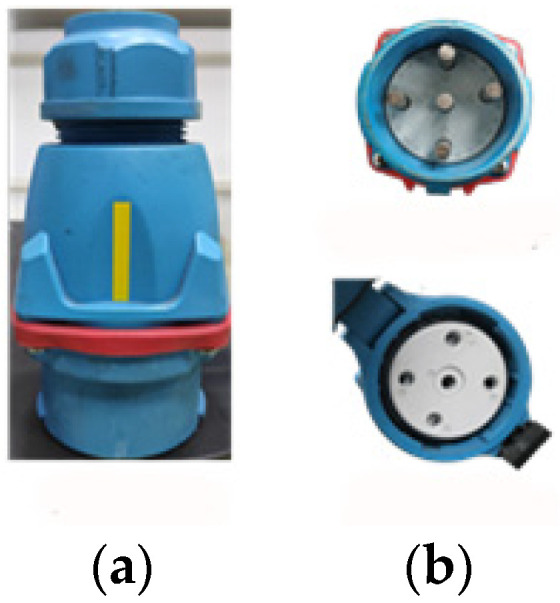
Example of highly axisymmetric properties. (**a**) Plug front view. (**b**) Plug and socket phase sequence.

To summarize, the target state estimation task, with lack of texture features, complex texture features, and high axisymmetric characteristics, is one of the difficult problems that needs to be solved at present [21]. In industrial applications, such as production lines, state estimation becomes particularly complex for industrial products with lack of texture features or high axisymmetric characteristics and strict requirements for multiple state angles. Figure 1 shows the example with the above characteristics, which is the object to be grasped by the manipulator arm. Figure 1a is a shore power plug for automatic docking. Figure 1b illustrates the axisymmetric characteristics of the shore power plug and the requirement for matching the phase sequence of a shore power plug and shore power socket.

For the target object with the above characteristics, a rectangular beacon (yellow beacon in Figure 1a) is designed in this paper. A deep binocular camera is adopted to extract the straight–line features of the beacon, based on the thinning process and Hough transform, combining the characteristics of the straight line (better robustness, anti–light, and anti–noise capabilities than the feature points). The state of the target object is solved analytically through the view relation of the linear beacon, and the state angle around the symmetry axis is included.

## 2. Extraction of Beacon Linear Features

According to the imaging model of a pinhole camera, the spatial line is still a straight line after the projection transformation. The premise of state estimation is to extract straight–line features representing rectangular beacons from two-dimensional images. The target object may have multiple linear features (contour, edge, etc.), so the rectangular beacon should be segmented from the background before the linear detection algorithm is applied. Image segmentation methods based on edges, regions, or contours are greatly affected by the texture features of actual images, so it is often necessary to adopt appropriate segmentation algorithms for specific objects.

Segmentation algorithms based on grayscale and color features are widely used in the field of machine vision. For the grayscale image of the source image $I_{traget}$ or the tonal channel image I in HSV space, the binary image B can be obtained by using thresholds $T_{1}$ and $T_{2}$ for segmentation [22]:

(1) $$B\left(x,\;y\right)=\{\begin{array}{l} 255,\;\;T_{1}\leq I\left(x,\;y\right)\leq T_{2}\\ 0 \end{array}$$

In the formula, B(x, y) and I(x, y) are pixel values of the column x, row y, of image B and image I, respectively.

Considering the beacon linear features, the method based on edge features or contour features will be affected by interference. Based on clusters, this method can suppress interference, but the robustness of the algorithm is not good, because the number of categories cannot be known in advance. The above methods do not consider the constraints of the beacon itself, so the beacon linear features cannot be extracted accurately.

In this paper, a beacon feature extraction method based on the thinning process and Hough line detection algorithm is proposed. By using the linear constraints existing in the beacon, the interference can be eliminated to a large extent.

The image thinning process is an algorithm that replaces the connected area of an image with a curve. In the thinning process, the boundary pixels are iteratively removed to form an image skeleton with a single–pixel width on the basis of maintaining the connectivity of the target. Zhang-Suen [23] algorithm is a parallel and 8-adjacency thinning algorithm. The most prominent advantage of Zhang-Suen algorithm is that the skeleton obtained after thinning is basically consistent with the original image for the areas such as lines, corners, and the intersection of T rows. Moreover, in terms of the complexity of the algorithm, it has fewer iterations and a fast running speed. According to the idea and characteristics of the image thinning algorithm, the thinned binary image T can be obtained by applying the algorithm to the above binary image  B.

Hough transform [24] converts curves in Euclidean space into points in parameter space, where a voting mechanism is used to detect the features of a given curve. For the binary image T, the progressive probabilistic Hough transform (PPHT) [25] can be applied to eliminate the influence of interference and obtain the straight–line segment describing the beacon. For the binary image obtained by color segmentation, PPHT is used to extract straight lines. The process is as follows:

(1) Random acquisition of pixel points whose value is not 0 in the binary image, according to the polar co-ordinate system linear equation: ρ=xcosθ+ysinθ, where ρ represents the distance from the normal line to the origin, and θ is the angle between the normal line and the polar co-ordinate axis. A two-dimensional (ρ, θ) point corresponds to a straight line, mapped onto a homogeneous two-dimensional co-ordinate curve of (ρ, θ), which represents all the straight lines of that pixel point.
(2) PPHT method is applied, where each pixel on its curve for (ρ, θ) is used to vote. If the number of (ρ, θ) points in the polar co-ordinate system reaches the minimum number of votes, the corresponding line L in the *x–y* co-ordinate system can be discovered.
(3) The points on the line L (and the distance between the points is less than the maximum distance set) are connected into line segments. Then, these points are all deleted and the parameters of the line segment are recorded (set starting point as S and end point as E). The length of the line segment should meet the minimum length condition.
(4) The above operations (1), (2), and (3) are repeated until all the pixels of the image have been traversed.

Using the line detection based on PPHT, n effective line segments can be obtained, and each line segment has the similar slope and two parameters, *S* and *E*, which are the starting point and end point, respectively. Then, the least square method is used to fit the 2n points into a straight line (obtaining the linear equation in pixel co-ordinates), that is, the extraction of beacon line features is completed.

## 3. State Estimation Based on the Beacon Linear Features and View Relation

In this chapter, the state of the target object is described mathematically, and the corresponding rotation matrix is established to solve the model. In addition, the state of the target object is estimated by combining the linear features mentioned in the previous chapter.

### 3.1. Target State Description

**Figure 2:**
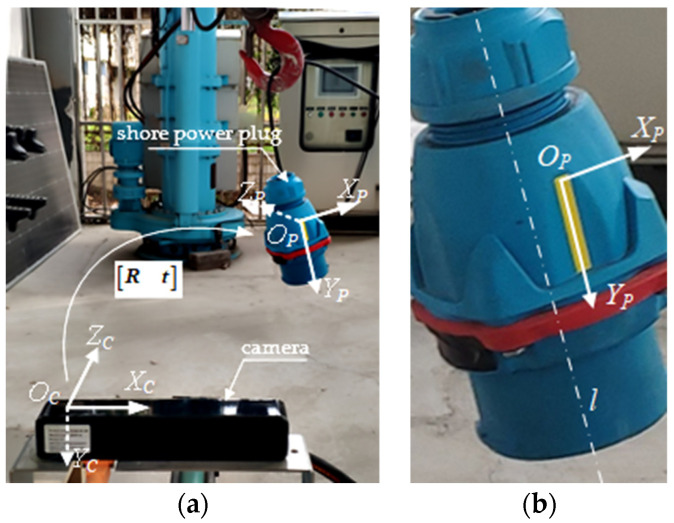
Camera and beacon co-ordinate system. (**a**) Relation between camera and beacon coordinate system. (**b**) Definition of the beacon coordinate system.

In the physical space, the object co-ordinate system $O_{P}-X_{P}Y_{P}Z_{P}$, its definition, and pose relation with the camera co-ordinate system $O_{C}-X_{C}Y_{C}Z_{C}$ are established with the rectangular beacon as the reference.$\;O_{P}-X_{P}Y_{P}Z_{P}$ has a pure translational relation with the co-ordinate system established at the origin of the target object’s centroid $C_{0}$, so the state of $O_{P}-X_{P}Y_{P}Z_{P}$ can be regarded as the state of the target object. Point $O_{P}$ is the midpoint of the short side above the rectangular beacon. co-ordinate axis $\vec{O_{P}X_{P}}$ is the short side direction of the beacon. co-ordinate axis $\vec{O_{P}Y_{P}}$ is the long side direction of the beacon. co-ordinate axis $\vec{O_{P}Z_{P}}$ is determined according to the right–hand rule and intersect with the central axis of the target object. The related co-ordinate systems are shown in Figure 2.

The transformation relationship of the vector between co-ordinate system $O_{P}-X_{P}Y_{P}Z_{P}$ and the camera co-ordinate system $O_{C}-X_{C}Y_{C}Z_{C}$ can be described as:

(2) $${\left[\begin{array}{ccc} x_{C} & y_{C} & z_{C} \end{array}\right]}^{T}=\left[\begin{array}{cc} R & t \end{array}\right]{\left[\begin{array}{cccc} x_{P} & y_{P} & z_{P} & 1 \end{array}\right]}^{T}$$

In the formula, $R=\left[\begin{array}{ccc} r_{1} & r_{2} & r_{3} \end{array}\right]$ is a 3 × 3 rotation matrix, t is a 3 × 1 translation matrix, and R is the state unknown.

If only the rotation matrix R is considered, the origin of the two co-ordinate systems can be coincident. In the case of the rotation matrix R, the geometric meaning is that the co-ordinate system $O_{C}-X_{C}Y_{C}Z_{C}$ goes through a series of rotations about a fixed axis (or the current axis), transforming to the co-ordinate system $O_{P}-X_{P}Y_{P}Z_{P}$. In general, $R=R_{z}\left(\varphi \right)R_{y}\left(\theta \right)R_{x}\left(\psi \right)$, and the working plane of the target object in most industrial applications is approximately parallel to the $X_{C}O_{C}Y_{C}$ plane of the camera, so the rotation about the $X_{C}$ axis can be ignored, i.e., $R_{x}\left(\psi \right)=I$. Therefore, the rotation matrix R is regarded as the composition of the rotation $R_{y}\left(\theta \right)$ about the axis $Y_{C}$ (fixed axis) and the rotation $R_{z}\left(\varphi \right)$ about the axis $Z_{C}$ (fixed axis), i.e.,:

(3) $$\;R=R_{z}\left(\varphi \right)R_{y}\left(\theta \right)$$

### 3.2. State–Solving Model Based on Rotation Matrix

#### 3.2.1. The Target Only Rotates *Ry*(θ ) about The *Y_C_* Axis

**Figure 3:**
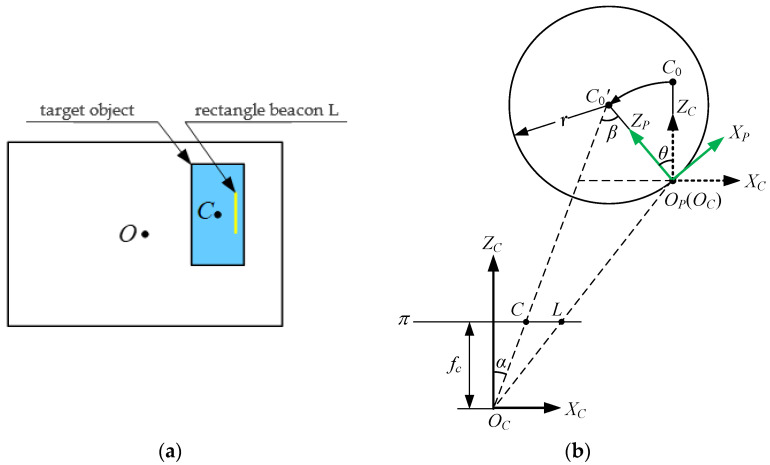
The projection and the $Y_{P}$ axis view of rotation only about the $Y_{C}$ axis. (**a**) The camera projects an image view as it rotates about the $Y_{C}$ axes. (**b**) $Y_{P}$ axial view.

If the target object only rotates $R_{y}\left(\theta \right)$ about the $Y_{C}$ axis, as shown in Figure 3, that is, the target object rotates angle *θ* about the $Y_{C}$ axis, its geometric center of mass rotates from $C_{0}$ to $C_{0}^{\prime }$, and its physical radius is r. The center point of the target object on the image plane π is C, and the co-ordinate of the corresponding point C in the camera co-ordinate system is $\left(x_{C},\;y_{C},\;z_{C}\right)$. Its value can be obtained after three-dimensional measurement. Line L can be obtained after the projection of the rectangular beacon is detected by the line.

According to the geometric relationship:

(4) $$rsin\beta =sd_{CL}cos\alpha$$

In the formula, $\alpha =arctan\frac{x_{C}}{z_{C}}$; $d_{CL}$ is the pixel distance from point C to line L; $s=\frac{z_{C}}{f_{c}}$ is the scaling factor, representing the physical length per pixel; and $\;f_{c}$ is the normalized focal length of the camera.

Therefore, in the figure ($x_{C}>0$, point C is to the left of line L), the rotation angle θ is:

(5) θ=α−β

In the same way, it can be obtained that θ=α+β when $x_{C}>0$ and point C is located on the right side of line L; θ=α−β when $x_{C}<0$ and point C is to the left of line L; θ=α+β when $x_{C}<0$ and point C is to the right of line L. Thus, the calculation model of rotation angle θ about the $Y_{C}$ axis can be uniformly expressed as:

(6)  θ=α+ζβ

In the formula, ζ=−1. when point C was to the left of line L, −1 when C was to the right of line L, and 0 when C was on line L. In addition, $\beta =arcsin[\frac{z_{C}d_{CL}cos\alpha }{\left(f_{c}\cdot r\right)}]$.

**Figure 4:**
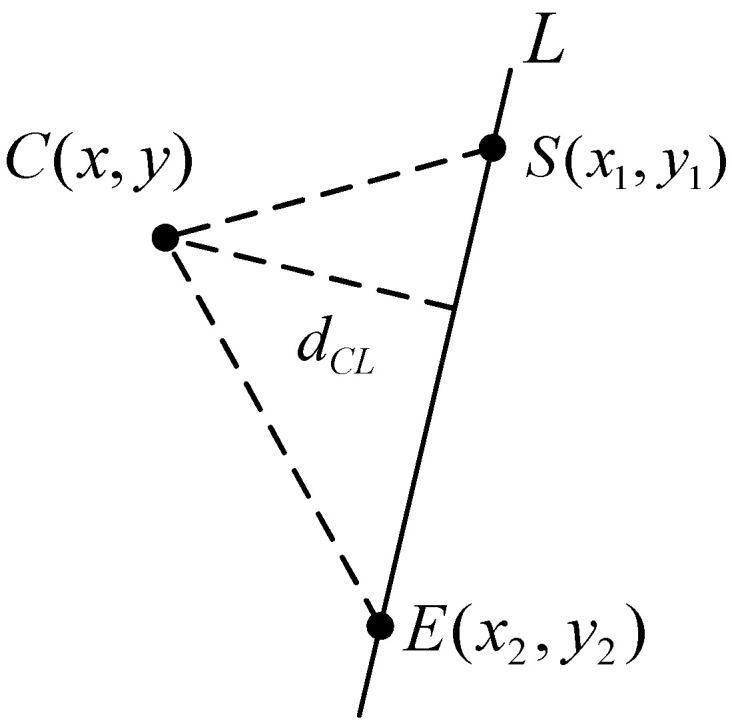
Schematic diagram of the relative position of the line and the point outside the line.

Line *L* and any two points on it, $S\left(x_{1},y_{1}\right)$, $E\left(x_{2},y_{2}\right)$, and the outer point C(x,y) are investigated on the pixel plane, as shown in Figure 4.

The pixel distance from point C to line L is $d_{CL}$:

(7) $$d_{CL}=\frac{2S_{\Delta SED}}{d_{SE}}$$

In the formula, $S_{\Delta SED}$ is the area of ΔSED, and $d_{SE}$ is the Euclidian distance between S and E.

The relative position relationship (left or right) between point C and line L can be represented by the scalar A, consisting of points $S\left(x_{1},y_{1}\right)$, $E\left(x_{2},y_{2}\right)$, and point C(x,y). When A>0, it means that point C(x,y) is on the left of line L, and when A<0, it means that point C(x,y) is on the right of line L. When A = 0, it means that the point C(x,y) lies on line L.

(8) $$A=\left(y_{2}-y_{1}\right)\left[\left(y-y_{1}\right)\left(x_{2}-x_{1}\right)-\left(y_{2}-y_{1}\right)\left(x-x_{1}\right)\right]$$

If the target object only rotates about the $Y_{C}$ axes, the rotation matrix R is:

(9) $$R=R_{y}\left(\theta \right)=\left[\begin{array}{ccc} cos\theta & 0 & sin\theta \\ 0 & 1 & 0\\ -sin\theta & 0 & cos\theta \end{array}\right]$$

#### 3.2.2. The Target Rotates about the *Z_C_* Axis and *Y_C_* Axis

**Figure 5:**
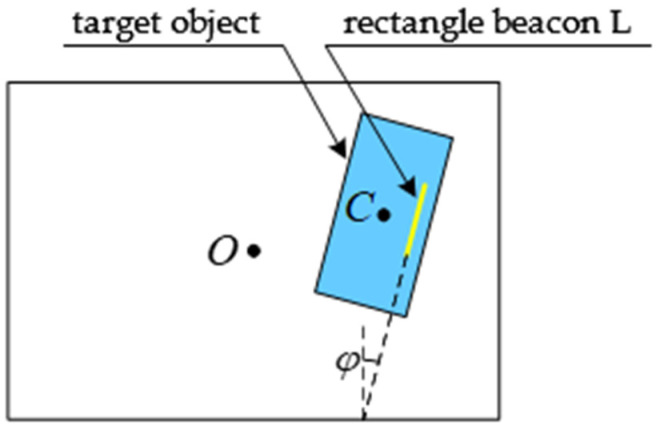
Camera projection image view as it rotates around the $Y_{C}$ and $Z_{C}$ axis.

The state R of the target object is regarded as the composition of the rotation $R_{y}$ about the $Y_{C}$ axis (fixed axis) and the rotation $R_{z}$ about the $Z_{C}$ axis (fixed axis). As shown in Figure 5, the rotation angle of the target object about the $Y_{C}$ axis is θ, and the rotation angle about the $Z_{C}$ axis is φ.

The angle of the projection line L of the rectangular beacon in the pixel plane represents the rotation angle φ about the $Z_{C}$ axis. As shown in Figure 4 and Figure 5, considering line L and any two points $S\left(x_{1},y_{1}\right)$ and $E\left(x_{2},y_{2}\right)$ on it, it is obvious that the rotation angle φ is:

(10) $$\varphi =arctan\left(\frac{x_{1}-x_{2}}{y_{1}-y_{2}}\right)$$

The rotation $R_{z}\left(\varphi \right)$ about the $Z_{C}$ axis (the fixed axis) is:

(11) $$R_{z}\left(\varphi \right)=\left[\begin{array}{ccc} cos\varphi & -sin\varphi & 0\\ sin\varphi & cos\varphi & 0\\ 0 & 0 & 1 \end{array}\right]$$

At this time, due:

(12) $${\left[\begin{array}{ccc} x_{C} & y_{C} & z_{C} \end{array}\right]}^{T}=R_{z}\left(\varphi \right){\left[\begin{array}{ccc} x_{C}^{\prime } & y_{C}^{\prime } & z_{C}^{\prime } \end{array}\right]}^{T}$$

In the formula, ${\left[\begin{array}{ccc} x_{C}^{\prime } & y_{C}^{\prime } & z_{C}^{\prime } \end{array}\right]}^{T}$ co-ordinate point is based on co-ordinate system $O_{C}-X_{C}^{\prime }Y_{C}^{\prime }Z_{C}^{\prime }$, which co-ordinate system $O_{C}-X_{C}Y_{C}Z_{C}$ transforms to by rotating φ about the $Z_{C}$–axis. It is obvious that $z_{C}=z_{C}^{\prime }$.

On this basis, the rotation $R_{y}\left(\theta \right)$ about the axis ${Y_{C}}^{\prime }$ (current axis) is further considered. Similar to the analysis process in the previous section, the calculation model of rotation angle θ is:

(13) θ=α+ζβ

In the formula, ζ=−1 when point C was to the left of line L, 1 when C was to the right of line L, and 0 when C was on line L.

(14) $$\{\begin{array}{l} {\left[\begin{array}{ccc} x_{C}^{\prime } & y_{C}^{\prime } & z_{C}^{\prime } \end{array}\right]}^{T}=R_{z}^{T}\left(\varphi \right){\left[\begin{array}{ccc} x_{C} & y_{C} & z_{C} \end{array}\right]}^{T}\\ \alpha =arctan\frac{x_{C}^{\prime }}{z_{C}^{\prime }}=arctan\frac{x_{C}cos\varphi +y_{C}sin\varphi }{z_{C}}\\ \beta =arcsin\left[\frac{z_{C}d_{CL}cos\alpha }{\left(f_{c}\cdot r\right)}\right] \end{array}$$

Thus, when the target object rotates about the $Y_{C}$ axis and then about the $Z_{C}$ axis, the rotation matrix is:

(15) $$R=R_{z}\left(\varphi \right)R_{y}\left(\theta \right)=\left[\begin{array}{ccc} cos\varphi & -sin\varphi & 0\\ sin\varphi & cos\varphi & 0\\ 0 & 0 & 1 \end{array}\right]\left[\begin{array}{ccc} cos\theta & 0 & sin\theta \\ 0 & 1 & 0\\ -sin\theta & 0 & cos\theta \end{array}\right]$$

## 4. Experiment and Result Analysis

In order to verify the accuracy and robustness of the algorithm, the linear feature extraction and state estimation were carried out with the shore power plug as the object. The shore power plug approximates a cylinder with highly axisymmetric characteristics, with a physical dimension of 176 mm high and an average diameter of d=86 mm in the middle section. The state estimation of the shore power plug is the premise of realizing the automatic docking between the shore power plug and the shore power socket by the manipulator arm. Since the phase sequence of the shore power plug and the shore power socket needs to match, it is necessary to detect its state around its axis l. The pose description between the shore power plug and the camera is shown in Figure 2. The yellow rectangular beacon in the figure is the straight–line feature to be extracted.

The YOLOV3 [26,27] target detection algorithm was used to identify and locate the shore power plug, and the depth camera was used to obtain the three-dimensional co-ordinates of the center point $\left(x_{C},\;y_{C},\;z_{C}\right)$.

### 4.1. Line Feature Extraction Experiment

**Figure 6:**
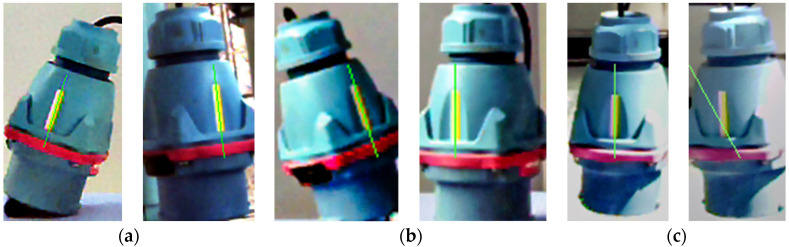
Beacon line feature extraction results. (**a**) weak light. (**b**) normal. (**c**) strong light.

The target object (the shore power plug) is randomly placed at any position at a random angle under different lighting backgrounds. Based on the target detection results, the beacon is divided on the hue channel of HSV space. Then, the extraction results of beacon linear features based on the thinning process and Hough transform proposed in this paper are shown in Figure 6.

**Figure 7:**
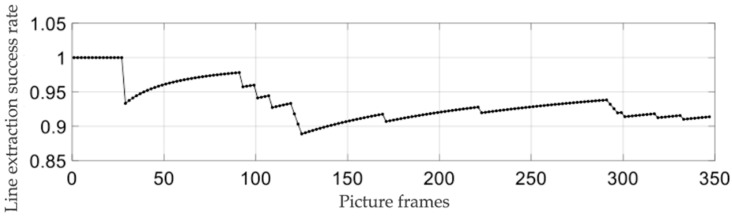
The relationship between line extraction success rate and frames.

The preliminary results show that the algorithm can detect the straight–line characteristics of the beacon in most conditions, but there are still some cases that some interference cannot be removed and leads to error detection. Further quantitative results are obtained to evaluate the specific performance of the algorithm correctly. Under the condition of different illumination, the target object (simple pendulum, translation) is shaken for continuous multiple frame detection, which means that the target object and rectangular beacon appear a variety of different angles. Under the condition of each kind of illumination and the different depth $z_{C}$, the experiment is repeated independently three times. Then, the statistical detection success rate $\eta_{L}$ is equal to $\frac{N_{L}}{N_{P}}$, where $N_{L}$ is the number of frames of successfully detecting line and $N_{P}$ is the number of frames of successfully detecting plug. In addition, the results are shown in Table 1. The total number of $N_{P}$ frames in each experiment is fixed as 300 frames. When the depth $z_{C}$ is equal to 790 mm under normal light, the relationship between the success rate and the number of frames is shown in Figure 7.

It can be seen from Table 1 that light intensity and depth distance have an influence on the success rate of beacon linear feature extraction. The general rule is that the success rate is negatively correlated with depth distance, and the success rate under the normal light condition is higher than that under weak light and strong light. It can be seen from Figure 7 that the success rate fluctuates with the increase in the number of frames, qualitatively indicating that the success rate of sample data is close to the overall success rate. The comprehensive success rate of beacon linear feature extraction is more than 80% by the experiment.

### 4.2. State Estimation Experiment and Analysis

In order to verify the accuracy of the proposed state estimation method (L–V method) based on the beacon linear feature and view relation, it is compared with the state estimation based on the beacon corner and homography matrix (C–H method) and the state estimation based on infinitesimal plane–based pose estimation (IPPE). Besides, in the experiment, it is found that the estimation value of C–H method is basically the same as that of another state estimation based on EPNP [28,29] (C–EPNP method).

**Figure 8:**
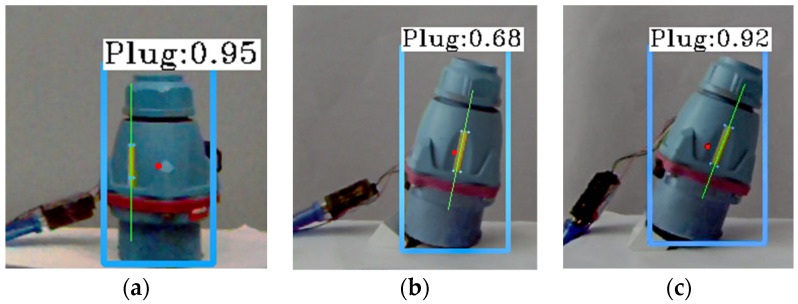
State measurement schematic diagram under three situations. (**a**) Rotate about $Y_{C}$ axis. (**b**) Rotate about $Z_{C}\;$ axis. (**c**) Rotate about $Y_{C}$ and $Z_{C}\;$ axis.

In the static scene, the target object is placed at random in different positions and attitudes, and the target object is rotated at angle θ only around the $Y_{C}$ axis, angle φ only around the $Z_{C}$ axis, and angle θ first around the $Y_{C}$ axis and then angle φ only around the $Z_{C}$ axis, respectively. A gyroscope is used to make objects present a different state, and the measured value of the gyroscope is taken as the true value. With continuous multiple frame detection of the target object, the linear feature extraction of failure and beacon corner detection frame are abandoned, and the data of the detection are averaged. Each experiment fixes the total effective frames $N_{R}=100$, under the condition of three kinds of detection diagram, as shown in Figure 8; blue box, blue dot, green line, and red dot represent target detection and its confidence, beacon corner, straight line feature, and center point detection results, respectively.

Regarding the description of the rotation state of the target object, there are various methods to describe the rotation state, and the commonly used methods are Euler angles [30] and quaternion [31]. Among them, the quaternion state description method tends to use numbers with certain calculation rules to describe the rotation state of the target object, which can be displayed intuitively through the certain calculation. The state description based on Euler angles is obtained by rotating three angles about three co-ordinate axes, and it is more intuitive. When Euler angles are used to describe the state in a three-dimensional space, the target object may fall into the problem of the gimbal lock [32]. However, according to the actual situation, the rotation angle of the target object around the camera’s co-ordinate axis $X_{c}$ is so small that it is ignored. Therefore, in this paper, the state of the target is described by Euler angles without the problem of the gimbal lock. In addition, considering the intuitionistic state description and the fact that the state solving algorithm in this paper can directly solve the Euler angles of the target object relative to the camera co-ordinate system, the Euler angles are used to express the state of the target more suitably than quaternion. The measurement results expressed by the RPY angle are shown in Table 2, Table 3 and Table 4, respectively.

(a) Only rotate θ about $Y_{C}$ axis

As can be seen from Table 2, the C–H method is unable to detect the changes caused by the rotation of the target object around the $Y_{C}$ axis, and the detection result is close to 0, that is, it is insensitive to the rotation around the $Y_{C}$ axis. The C–IPPE method is very inaccurate in the detection of angle θ. The absolute value of relative error $e_{L–V}\;$ is less than 9%, the absolute value of absolute error is less than 3°, and the average absolute percentage error is 3.711.

(b) Only rotate φ about $Z_{C}$ axis

As can be seen from the results in Table 3, the measurement error of the L–V method for the rotation angle φ is not more than 10%, and the absolute value of the absolute error is not more than 2°. At the same time, it is better than the C–H method and C–IPPE method. The C–H method and C–IPPE method can perceive the change of φ, and have better measurement results.

(c) Rotation angle (θ, φ) about the $Y_{C}$ axis and then about the $Z_{C}$ axis

In Table 4, the C–H method and C–IPPE method are still insensitive to the change of θ. In Table 5, the relative error percentage and average absolute percentage error $e_{\mathrm{MAPE}}$ of each method in Table 4 are calculated, but the detection error of θ by the C–H method and C–IPPE method is not included.

By comparison with Table 2 and Table 5, for the L–V method, the detection accuracy of θ is reduced due to the presence of φ. The L–V method is still superior to the other two methods; the absolute value of relative error is less than 15%, the absolute value of absolute error is less than 5°, and the average absolute percentage error is 9.132%.

**Figure 9:**
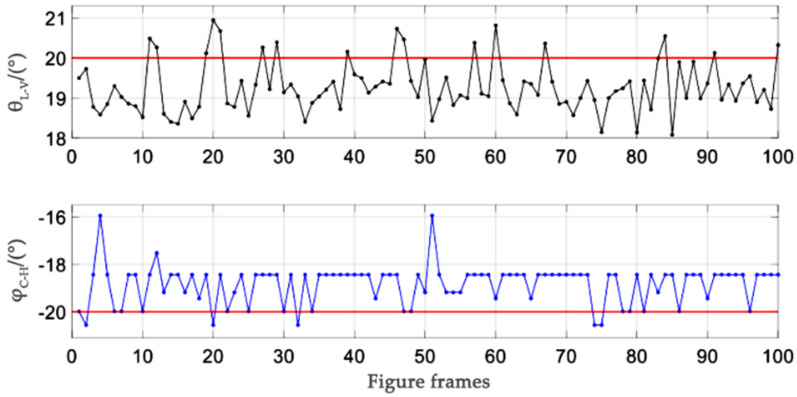
*θ* = 20°, *φ* = −20° detection value respectively through L–V and C–H methods.

Taking the experiment numbered 4 in Experiment (c) as an example, i.e., θ=20° and φ= −20°, the detection value of 100 frames of images is shown in Figure 9. $\theta_{L–V}$ is the detection result of the LV method, and $\varphi_{C–H}$ is the detection result of the C–H method.

As can be seen from Figure 9, the state detection results of the L–V method and C–H method are relatively stable, fluctuating around the true value, and the difference of the peak value is within 4°.

In the subsequent work, this paper increased the experimental valid frame *n* to 1000 frames. The experimental results are compared with those of *n* = 100 frames, and there is little difference between them. In addition, it is tested that this method can complete the grasping and docking of the shore power plug with the manipulator in the shore power docking experiment. 

The reason why the C–H method cannot achieve perception of the rotation angle θ about the fixed axis $Y_{C}$ is mainly as follows: weak perspective projection problems of the beacon, namely the similar depth of the beacon at the four corners, causing a failure of the homography matrix on θ state estimation. The L–V method in this paper realized the detection of θ and a more accurate and stable detection of the rotation angle φ about axis $Z_{C}$, namely to achieve state estimation of an axisymmetric target.

## 5. Conclusions

Aiming at a target that lacks texture and has the characteristics of axial symmetry, in this paper, we add bright–colored straight–line beacons to the target artificially, and successfully extract the straight–line features of the beacons through the Hough transform and thinning process. Finally, the state estimation of a highly axisymmetric target, including the state angle about its axis of symmetry, is realized by using the features of a single linear and view relation. In addition, through the comparison of the success rate of line extraction under different illumination and image frame numbers, the success rate of line extraction is maintained at about 80%, which indicates that the robustness of line feature extraction is strong. Therefore, the reason why the subsequent C–H and C–IPPE methods can not accurately estimate *θ* is that the anti–interference ability of corner points is weaker than that of straight lines, and the robustness is poor. By comparing the experimental data measured by the L–V method, C–H method, and C–IPPE method, it is shown that the L–V method is not only more accurate than the latter two methods in estimating the state angle *φ*, but also can more accurately estimate the state angle *θ* at which the highly axisymmetric target rotates about the axis of symmetry. In summary, it is verified that the L–V method can estimate the state of axisymmetric targets with higher accuracy and robustness.

## 6. Future Work

As for the future research of this topic, in order to reduce environmental interference and improve the feasibility of application, it is necessary to combine this algorithm with a deep–learning network, which can identify and capture target objects in the image, so as to reduce the interference of the external complex environment. Compared with the deep–learning network that directly trains and estimates the target state through images, the deep–learning network for target recognition is more mature, and its training set and network complexity are simpler. In addition, the state estimation in this paper takes into account the factor of the small rotation angle of a shore power plug around the $X_{C}$ axis in the actual situation, and ignores it. In order to expand its application range and improve the accuracy of state estimation, its rotation angle around the $X_{c}$ axis still needs to be discussed in 3D space. The Euler angles of the axisymmetric object rotating about its own co-ordinate axis are used as its attitude description, and its rotation order is *X–Y–Z*. According to this rotation order, rotation about the *Y*–axis only changes the length projection of the target object on its *Z*–axis, and has no effect on the *θ* and *φ* angle solving method mentioned in this paper. Then, the angle of rotation around the *Y*–axis can be calculated according to the change in the length of its beacon projection. Since there are no experiments, we are simply making assumptions here.

## Figures and Tables

**Table 1:** Success rate of beacon straight–line feature extraction under different conditions.

Condition	Weak Light	Normal Light	Strong Light
$z_{C}=480\;\mathrm{mm}$	96.00%	98.14%	98.33%
$z_{C}=790\;\mathrm{mm}$	77.33%	91.38%	74.67%
$z_{C}=1100\;\mathrm{mm}$	58.75%	64.86%	59.00%
Average	77.36%	84.79%	77.33%

**Table 2:** Measurement results of rotation angle θ about the $Y_{C}$ axis.

True–Value $\theta_{r}\left({}^{\circ}\right)$	$\theta_{L–V}\left({}^{\circ}\right)$	$e_{L–V}\left(\%\right)$	$\theta_{C–H}\left({}^{\circ}\right)$	$\theta_{C–IPPE}\left({}^{\circ}\right)$
10	10.837	8.37	0	3.368
−10	−10.220	2.20	−0.004	−31.023
20	19.126	−4.37	0.008	15.538
−20	−20.287	1.44	0	−10.447
30	29.521	−1.60	−0.002	9.903
−30	−31.774	5.91	0	−17.143
40	41.549	3.87	0	34.355
−40	−37.445	−6.39	0	−9.613
50	48.585	−2.83	0	26.053
−50	−49.933	−0.13	0	0

**Table 3:** Measurement results of rotation angle φ about the $Z_{C}$ axis.

$\mathrm{True}–\mathrm{Value}\;\varphi_{r}\left({}^{\circ}\right)$	$\varphi_{L–V}\left({}^{\circ}\right)$	$e_{L–V}\left(\%\right)$	$\varphi_{C–H}\left({}^{\circ}\right)$	$e_{C–H}\left(\%\right)$	$\varphi_{C–\mathrm{IPPE}}\left({}^{\circ}\right)$	$e_{C–IPPE}\left(\%\right)$
10	10.690	6.90	10.893	8.93	8.364	−16.36
−10	−10.055	0.55	−10.420	4.20	−11.277	12.77
20	21.245	6.23	23.324	16.62	17.562	−12.19
−20	−21.902	9.51	−22.201	11.01	−22.436	12.18
30	30.586	1.95	31.890	6.30	27.156	−9.48
−30	−30.695	2.32	−31.854	6.18	−32.697	8.99

**Table 4:** Measurement results of rotation angle (θ, φ) around the $Y_{C}$ axis and then around then $Z_{C}$ axis.

Number	True–Value	L–V	C–H	C–IPPE
1	(0, 0)	(1.809, −1.716)	(0, 0)	(1.954, −0.306)
2	(10, −10)	(8.574, −10.997)	(0.002, −11.160)	(−7.772, −9.972)
3	(−10, 10)	(−9.922, 11.275)	(−0.006, 11.717)	(−1.680, 12.052)
4	(20, −20)	(19.281, −18.633)	(0.001, −18.823)	(−8.735, −17.86)
5	(−20, 20)	(−21.357, 20.311)	(0.007, 20.555)	(−3.137, 21.256)
6	(30, −30)	(25.617, −28.421)	(0.001, −26.565)	(5.394, −26.909)
7	(−30, 30)	(−25.576, 28.086)	(0, 27.416)	(0, 25.713)

**Table 5:** Measurement error of (θ, φ).

Number	$e_{L–V}\left(\%\right)\left(\theta \right)$	$e_{L–V}\left(\%\right)\left(\varphi \right)$	$e_{C–H}\left(\%\right)\left(\varphi \right)$	$e_{C–\mathrm{IPPE}}\left(\%\right)\left(\varphi \right)$
2	−14.26	9.97	11.60	−0.28
3	−0.78	12.75	17.17	20.52
4	−3.60	−6.84	−5.89	−10.67
5	6.79	1.56	2.78	6.28
6	−14.61	−5.26	−11.45	−10.30
7	−14.75	−6.38	−8.61	−14.29
$e_{MAPE}$	9.132	7.127	9.583	10.390

## Data Availability

Not applicable.
